# Leukotriene biosynthesis inhibition ameliorates acute lung injury following hemorrhagic shock in rats

**DOI:** 10.1186/1749-8090-6-81

**Published:** 2011-06-07

**Authors:** Fadhil G Al-Amran, Najah R Hadi, Ali M Hashim

**Affiliations:** 1 Department of Surgery, Colorado Denver university, Box C-320 12700 E 19th Avenue, Aurora, CO 80045 USA; 2 Department of pharmacy, Kufa university, Najaf kufa street, Najaf, Iraq

**Keywords:** MK-886, hemorrhagic shock, acute lung injury, oxidative stress, inflammatory markers

## Abstract

**Background:**

Hemorrhagic shock followed by resuscitation is conceived as an insult frequently induces a systemic inflammatory response syndrome and oxidative stress that results in multiple-organ dysfunction syndrome including acute lung injury. MK-886 is a leukotriene biosynthesis inhibitor exerts an anti inflammatory and antioxidant activity.

**Objectives:**

The objective of present study was to assess the possible protective effect of MK-886 against hemorrhagic shock-induced acute lung injury via interfering with inflammatory and oxidative pathways.

**Materials and methods:**

Eighteen adult Albino rats were assigned to three groups each containing six rats: group I, sham group, rats underwent all surgical instrumentation but neither hemorrhagic shock nor resuscitation was done; group II, Rats underwent hemorrhagic shock (HS) for 1 hr then resuscitated with Ringer's lactate (1 hr) (induced untreated group, HS); group III, HS + MK-886 (0.6 mg/kg i.p. injection 30 min before the induction of HS, and the same dose was repeated just before reperfusion period). At the end of experiment (2 hr after completion of resuscitation), blood samples were collected for measurement of serum tumor necrosis factor-α (TNF-α) and interleukin-6 (IL-6). The trachea was then isolated and bronchoalveolar lavage fluid (BALF) was carried out for measurement of leukotriene B_4 _(LTB_4_), leukotriene C_4 _(LTC_4_) and total protein. The lungs were harvested, excised and the left lung was homogenized for measurement of malondialdehyde (MDA) and reduced glutathione (GSH) and the right lung was fixed in 10% formalin for histological examination.

**Results:**

MK-886 treatment significantly reduced the total lung injury score compared with the HS group (*P *< 0.05). MK-886 also significantly decreased serum TNF-α & IL-6; lung MDA; BALF LTB_4_, LTC_4 _& total protein compared with the HS group (*P *< 0.05). MK-886 treatment significantly prevented the decrease in the lung GSH levels compared with the HS group (*P *< 0.05).

**Conclusions:**

The results of the present study reveal that MK-886 may ameliorate lung injury in shocked rats via interfering with inflammatory and oxidative pathways implicating the role of leukotrienes in the pathogenesis of hemorrhagic shock-induced lung inflammation.

## 1. Introduction

Hemorrhagic shock (HS) is a commonly encountered complication within a blunt traumatic or surgical injury. Hemorrhagic shock followed by resuscitation (HSR) is conceived as an insult frequently induces a systemic inflammatory response syndrome (SIRS) that results in multiple-organ dysfunction syndrome (MODS) [1,2]including acute lung injury (ALI), which is a major clinical problem, leading to significant mortality and morbidity [1,3]. The mechanism of pathogenesis of SIRS in the field of HS is complex and a variety of mechanisms are implicated. The most widely recognized mechanisms are ischemia and reperfusion (I/R) and stimulation of cells of the innate immune system [4]. Ischemia and reperfusion is mainly participating in oxidative stress and SIRS arising during post-ischemic resuscitation. I/R injury is, by itself, a potent inflammatory trigger, increasing cytokine release, reactive oxygen species generation, and endothelial activation, with consequent nitric oxide production and expression of adhesion molecules [5]. Neutrophils are the major cellular elements involved in acute lung inflammation after resuscitated hemorrhagic shock [6]. Studies have shown that neutrophils are activated following HS [7] and that lung injury is associated with an increased neutrophils accumulation in the lungs after HS [8]. The activated neutrophils appear to infiltrate the injured lung in parallel with increased expression of adhesion molecules on endothelial cells and elevated local chemokines/cytokines levels following HS [7].

MK-886 (investigational compound) is a highly potent inhibitor of leukotriene formation in vivo and in vitro [9]. This compound inhibits leukotriene biosynthesis indirectly by a mechanism through the binding of a membrane bound 5-lipoxygenase-activating protein (FLAP), thereby inhibiting the translocation and activation of 5-lipoxygenase [10,11]. The 5-lipoxygenase inhibition by MK-886 prevents stimulated neutrophil adherence and chemotaxis and neutrophil mediated lung injury in vitro [12]. MK-886 has been shown to reduce the extravasation of plasma [13] and prevent the leukocyte adhesion to the endothelium [14] in experimental animals. MK-886 was found to be effective in prevention of liver and intestine injury by reducing apoptosis and oxidative stress in a hepatic I/R model. Anti-inflammatory properties and inhibition of lipid peroxidation by MK-886 could be protective for these organs in I/R injury [15] . MK-886 significantly reduces acute colonic mucosal inflammation in animals with colitis when the treatment is performed during the early phase of the inflammatory response [16] . Recently, treatment of mice with MK-886 significantly abolished the increase in the BALF total protein level in a model of acute lung injury following hemorrhagic shock [17].

## 2. Materials and methods

### 2.1. Animals and Study Design

A total of eighteen adult male Albino rats weighing 150-220 g were purchased from Animal Resource Center, the Institute of embryo research and treatment of infertility, Al-Nahrain University. They were housed in the animal house of Kufa College of Medicine in a temperature-controlled (25°C) room with alternating 12-h light/12-h dark cycles and were allowed free access to water and chow diet until the start of experiments. All experiments were approved by the Animal Care and Research Committee of the University of Colorado Denver, and this investigation conforms with the Guide for the Care and Use of Laboratory Animals (National Research Council, revised 1996).

After the 1^st ^week of acclimatization the rats were randomized into three groups as follow:

I. **Sham group**: this group consisted of 6 rats; rats underwent the same anesthetic and surgical procedures for an identical period of time as shock animals, but neither hemorrhage nor fluid resuscitation was performed.

II. **Control group**: (induced untreated group): this group consisted of six rats; rats underwent hemorrhagic shock (for 1 hr) then resuscitated with Ringer's lactate (RL) (for 1 hr), and left until the end of the experiment.

III. **MK-886 treated group**: this group consisted of 6 rats; Rats received MK-886 0.6 mg/kg i.p. injection 30 min before the induction of HS, and the same dose was repeated just before reperfusion period.

❖Both sham and induced untreated rats received the same volume of the vehicle.

The drug was purchased from (Cayman chemical, USA) and prepared immediately before use as a homogenized solution in 2% ethanol [15] . Ethanol was used to form a homogenized drug. Each dose was homogenized in 1ml ethanol and injected via i.p [15].

### 2.2. Hemorrhagic Shock Protocol

Animals were intraperitoneally anesthetized with 80 mg/kg ketamine and 8 mg/kg xylazine [18] and subjected to a 50% blood loss (30 ml/kg) via intracardiac puncture from the left side of the chest over 2 min and left in shock state for 1 hr. The animals were then resuscitated with two times blood loss (60 ml/kg) using i.v lactated Ringers via tail over 1 hr [19]^.^The sham group underwent all instrumentation procedures, but neither hemorrhage nor resuscitation was carried out. Animals were allowed to breathe spontaneously throughout the experiment. Two hour after the completion of resuscitation, rats were again anesthetized and sacrificed by exsanguinations, where the chest cavity was opened and blood samples were taken directly from the heart. The trachea was then isolated and bronchoalveolar lavage fluid (BALF) was carried out. The lungs were harvested, excised and the left lung was homogenized and stored until use for the study and the right lung was fixed in 10% formalin for histological examination.

### 2.3. Preparation of Blood Samples and Cytokine Assays

About 3 ml of blood was collected from the heart of each rat. The blood sampling was done at the end of the experiment (2hr after the completion of resuscitation). The blood samples were allowed to clot at 37°C and then centrifuged at 3000 rpm for 15 min; Sera were removed, and analyzed for determination of serum TNF-a and IL-6. Serum TNF-a and IL-6 were quantified according to the manufacturer's instructions and guidelines using enzyme-linked immunosorbent assay (ELISA) kits (IMMUNOTECH. France).

### 2.4. Preparation of Bronchoalveolar Lavage Fluid and determination of leukotrienes and total protein

The trachea was then isolated, and bronchoalveolar lavage fluid was obtained by washing the airways four times with 5 ml of phosphate buffered saline. The bronchoalveolar lavage fluid was centrifuged at 1200 × *g *for 10 min at 4°C. The supernatant was collected and stored at -70°C until analyzed for LTB_4_, LTC_4 _and total protein [20]. The BALF levels of LTB_4 _and LTC_4 _were quantified according to the manufacturer's instructions and guidelines using ELISA kits (USBiological. USA). Cell free BALF was evaluated for total protein content using Biuret method (photometric colorimetric test total proteins) [21].

### 2.5. Tissue Preparation for Oxidative Stress Measurement

The lung specimens were homogenized with a high intensity ultrasonic liquid processor and sonicated in phosphate buffered saline containing 0.1mmol/L EDTA (pH7.4) (10%). The homogenate was centrifuged at 10 000 rpm for 15 min at 4°C and supernatant was used for determination of GSH and MDA [18] . The MDA levels were assayed for products of lipid peroxidation by monitoring thiobarbituric acid reactive substance formation according to the method of Buege and Aust in 1978 [22]. Lipid peroxidation was expressed in terms of MDA equivalents using an extinction coefficient of 1.56 × 10^5 ^M^−1 ^cm ^−1 ^and results were expressed as nmol MDA/g tissue. GSH measurements were performed using a colorimetric method at 412nm (BioAssay Systems' QuantiChrom™ Glutathione Assay Kit).

### 2.6. Tissue Sampling for Histopathology

At the end of the experiment, rats were sacrificed and the lung was harvested. All specimens were immediately fixed in 10% buffered formalin. After fixation they were processed in usual manner. The sections were examined by microscope then the histological changes were determined.

The degree of lung injury was assessed using the scoring system described by **Matute-Bello *et al. ***that graded congestion of alveolar septae, intra-alveolar cell infiltrates, and alveolar hemorrhage [23]. Each parameter was graded on a scale of 0-3, as follows: alveolar septae, 0: septae thin and delicate, 1: congested alveolar septae in < 1/3 of the field, 2: congested alveolar septae in 1/3-2/3 of the field, 3: congested alveolar septae in > 2/3 of the field; intra-alveolar cell infiltrates, 0: < 5 intra-alveolar cells per field, 1: 5 to 10 intra-alveolar cells per field, 2: 10 to 20 intra-alveolar cells per field, 3: > 20 intra-alveolar cells per field; Alveolar hemorrhage, 0: no hemorrhage, 1: at least 5 erythrocytes per alveolus in 1 to 5 alveoli, 2: at least 5 erythrocytes in 5 to 10 alveoli, 3: at least 5 erythrocytes in > 10 alveoli. The total lung injury score was calculated be adding the individual scores for each category and lung injury was categorized according to the sum of the score to normal (0), mild (1-3), moderate (4-6) and severe injury (7-9). The histological sections were evaluated by a pathologist without prior knowledge of the treatment given to the animals.

### 2.7. Statistical Analysis

Statistical analyses were performed using SPSS 12.0 for windows.lnc. Data were expressed as mean ± SEM. Analysis of Variance (ANOVA) was used for the multiple comparisons among all groups followed by post-hoc tests using LSD method. The histopathological grading of lung changes is a non-normally distributed variable measured on an ordinal level of measurement; therefore non-parametric tests were used to assess the statistical significance involving this variable. The statistical significance of difference in total score between more than 2 groups was assessed by Kruskal-Wallis test, while Mann-Whitney U test was used for the difference between 2 groups. In all tests, P < 0.05 was considered to be statistically significant.

## 3. Results

### 3.1. Effect on Proinflammatory Cytokines (TNF-α and IL-6)

**Figure 1:**
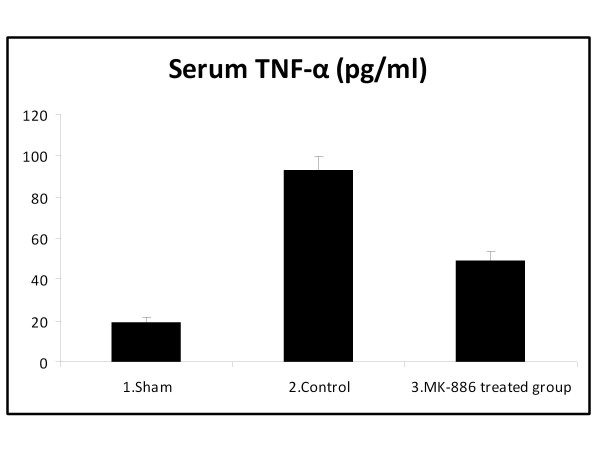
**The mean of serum TNF-α level (pg/ml) in the three experimental groups at the end of the experiment**.

**Figure 2:**
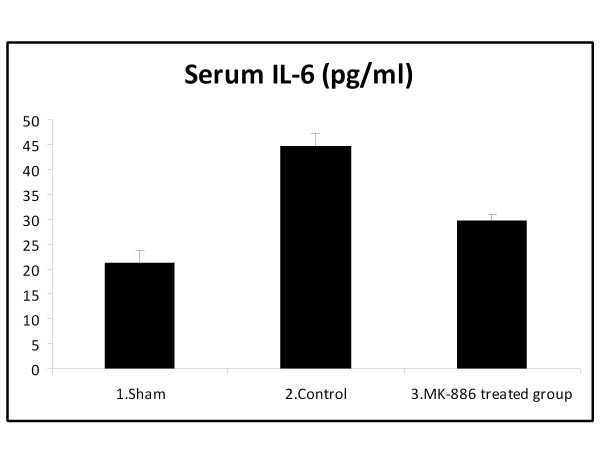
**The mean of serum IL-6 level (pg/ml) in the three experimental groups at the end of the experiment**.

At the end of the experiment, the serum TNF-α and IL-6 levels were significantly higher in the HS group when compared with the sham group (*P *< 0.05). Treatment with MK-886 significantly decreased the serum TNF-α and IL-6 levels when compared with the HS group (*P *< 0.05). The TNF-α and IL-6 values for the different groups are shown in table 1 and Figures 1&2.

**Table 1:** Serum TNF-α and IL-6 levels (pg/ml) of the three experimental groups at the end of the experiment

Group	TNF-α (pg/ml)	IL-6 (pg/ml)
1. Sham	19.4 ± 2.12	21.16 ± 2.61

2. Control (HS)	93.3 ± 6.48*	44.84 ± 2.33*

3. MK-886 treated group	49.4 ± 3.81^†^	29.78 ± 1.27^†^

The data expressed as mean ± SEM (N = 6 in each group).

• *P *< 0.05 vs. sham group, ^† ^*P *< 0.05 vs. HS (induced untreated) group

### 3.2. Effect on Lung MDA and GSH Levels

**Figure 3:**
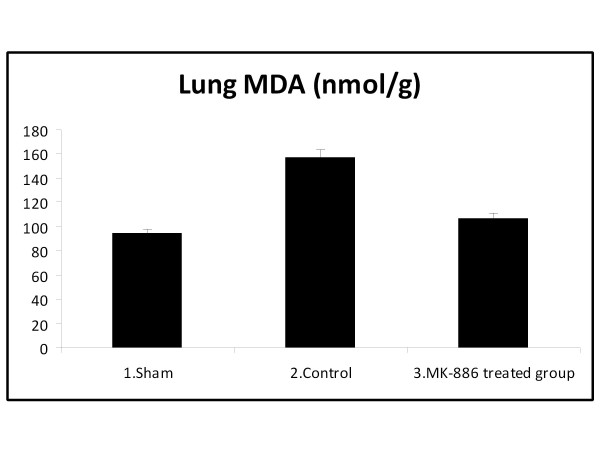
**The mean of lung MDA level (nmol/g) in the three experimental groups at the end of the experiment**.

**Figure 4:**
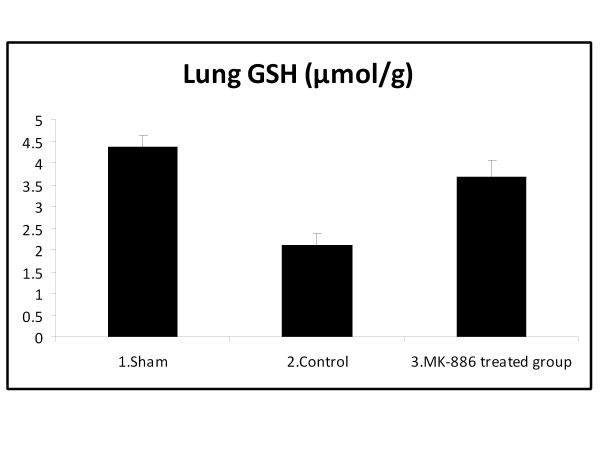
**The mean of lung GSH level (μmol/g) in the three experimental groups at the end of the experiment**.

The MDA levels, measured as a major degradation product of lipid peroxidation in the pulmonary tissue, were found to be significantly higher in HS group as compared to those of the sham group (*P *< 0.05), while treatment with MK-886 abolished these elevations (*P *< 0.05). The HS caused a significant decrease in lung GSH level (*P *< 0.05) when compared with the sham group, while in the MK-886 treated group, the lung GSH level was found to be preserved (*P *< 0.05) and not significantly different from that of the sham group. The MDA and GSH values for the different groups are shown in table 2 and Figure 3, 4.

**Table 2:** Lung MDA and GSH levels of the three experimental groups at the end of the experiment

Group	Lung MDA (nmol/g)	Lung GSH (μmol/g)
1. Sham	95 ± 2.78	4.36 ± 0.27

2. Control (HS)	157 ± 6.15*	2.12 ± 0.25*

3. MK-886 treated group	107.2 ± 3.76^†^	3.7 ± 0.35^†^

The data expressed as mean ± SEM (N = 6 in each group).

• *P *< 0.05 vs. sham group, ^† ^*P *< 0.05 vs. HS (induced untreated) group

### 3.3. Effect on Leukotrienes (LTB_4 _& LTC_4_)

**Figure 5:**
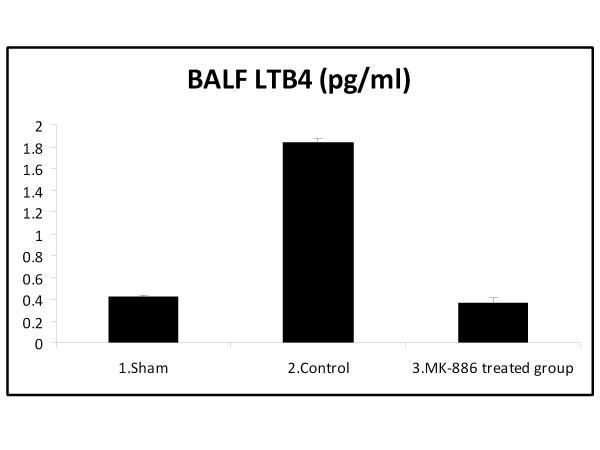
**The mean of BALF LTB_4 _level (pg/ml) in the three experimental groups at the end of the experiment**.

**Figure 6:**
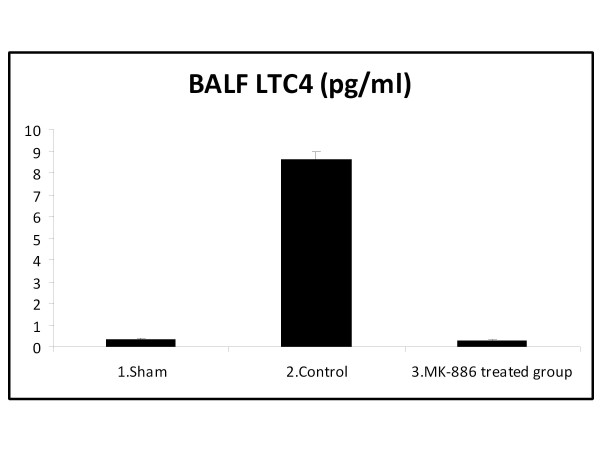
**The mean of BALF LTC_4 _level (pg/ml) in the three experimental groups at the end of the experiment**.

At the end of the experiment; the LTB_4 _and LTC_4 _levels in the BALF were significantly increased in the HS group as compared with the sham group (*P *< 0.05). Treatment with MK-886 significantly decreased the BALF LTB_4 _and LTC_4 _levels when compared with the HS group (*P *< 0.05). The LTB_4 _and LTC_4 _values for the different groups are shown in table 3 and Figure 5, 6.

**Table 3:** BALF LTB_4 _and LTC_4 _level (pg/ml) of the three experimental groups at the end of the experiment

Group	BALF LTB_4 _(pg/ml)	BALF LTC_4 _(pg/ml)
1. Sham	0.42 ± 0.02	0.33 ± 0.05

2. Control (HS)	1.84 ± 0.03*	8.64 ± 0.31*

3. MK-886 treated group	0.37 ± 0.04^†^	0.28 ± 0.05^†^

The data expressed as mean ± SEM (N = 6 in each group).

• *P *< 0.05 vs. sham group, ^† ^*P *< 0.05 vs. HS (induced untreated) group

### 3.4. Effect on BALF Total Protein

**Figure 7:**
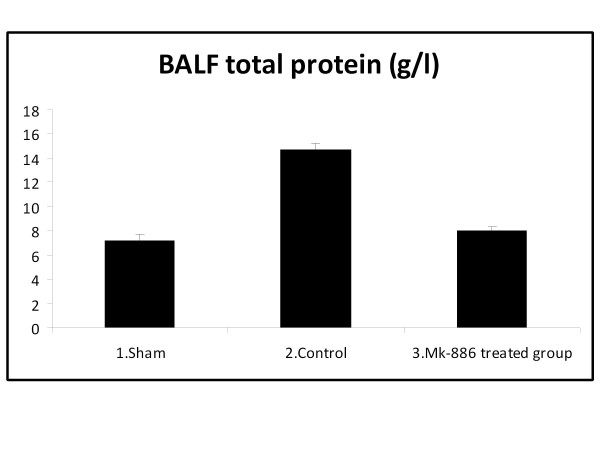
**The mean of BALF total protein level (g/l) in the three experimental groups at the end of the experiment**.

At the end of the experiment; the total protein level of the BALF was significantly increased in HS group as compared with sham group (*P *< 0.05). Treatment with MK-886 significantly decreased the BALF total protein levels when compared with the HS group (*P *< 0.05). The total protein values for the different groups are shown in table 4 and Figure 7.

**Table 4:** BALF total protein level (g/l) of the three experimental groups, at the end of the experiment

Group	BALF total protein (g/l)
1. Sham	7.2 ± 0.5

2. Control (HS)	14.7 ± 0.57*

3. MK-886 treated group	8 ± 0.3^†^

The data expressed as mean ± SEM (N = 6 in each group).

• *P *< 0.05 vs. sham group, ^† ^*P *< 0.05 vs. HS (induced untreated) group

### 3.5. Histological finding

**Figure 8:**
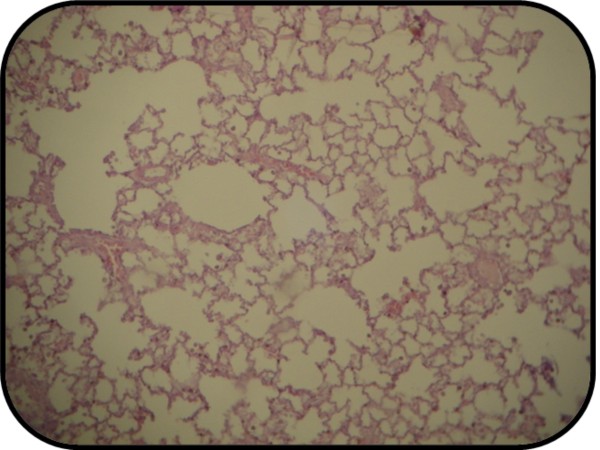
**Photomicrograph of lung section of normal rats shows the normal architecture**. The section stained with Haematoxylin and Eosin (X 10).

A cross section of sham rat's lung showed the normal appearance of all three parameters (thin and delicate alveolar septae, no intra-alveolar cell infiltrates and no alveolar hemorrhage) Figure 8. All rats in this group showed normal lung appearance (100%) as shown in table 5.

**Table 5:** The differences in histopathological grading of abnormal lung changes among the three experimental groups

Histopathological grading	Study group					
	
	Sham		Control (HS)		MK-886	
	
	N	%	N	%	N	%
**Normal**	6	100	0	0	1	16.7

**Mild**	0	0	0	0	5	83.3

**Moderate**	0	0	4	66.7	0	0

**Severe**	0	0	2	33.3	0	0

**Total**	6	100	6	100	6	100

**Figure 9:**
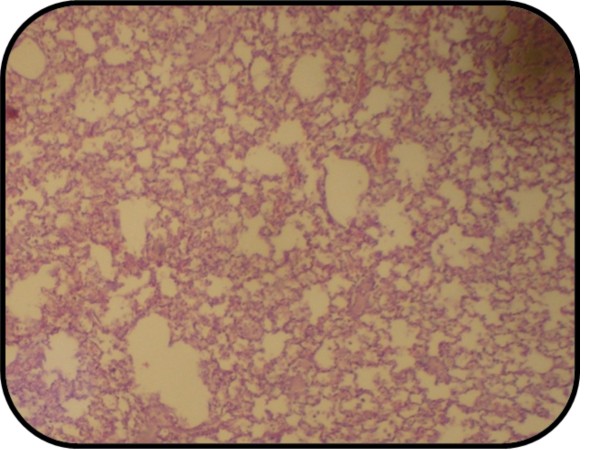
**Photomicrograph of lung section with moderate injury**. The section stained with Haematoxylin and Eosin (X 10).

**Figure 10:**
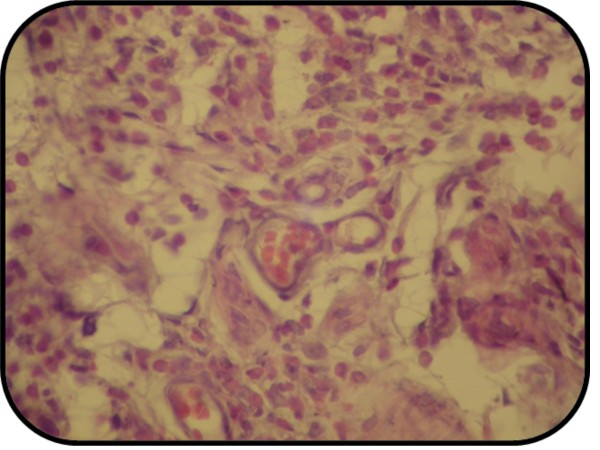
**Photomicrograph of lung section with severe injury**. The section stained with Haematoxylin and Eosin (X 40).

There was statistically significant difference between induced untreated (HS) group and sham group (*P *< 0.05) and the total score mean of the HS group showed moderate lung injury. 66.7% of the group had moderate lung injury and 33.3% had severe lung injury as shown in table 5, 6 and Figures 9, 10.

**Table 6:** Acute lung injury score

Study group	Congestion of alveolar septae	Intra-alveolar cell infiltrates	Alveolar hemorrhage	Total score	Total score grade
**Sham**	0	0	0	0	Normal

**HS**	1.5 ± 0.34	2.5 ± 0.22	1.83 ± 0.16	5.83 ± 0.60*	Moderate

**MK-886 treated group**	0.5 ± 0.22	0.66 ± 0.21	0.17 ± 0.16	1.33 ± 0.42^†^	Mild

The data expressed as means ± SEM.

* *P *< 0.05 vs. sham group, ^† ^*P *< 0.05 vs. HS (induced untreated) group

**Figure 11:**
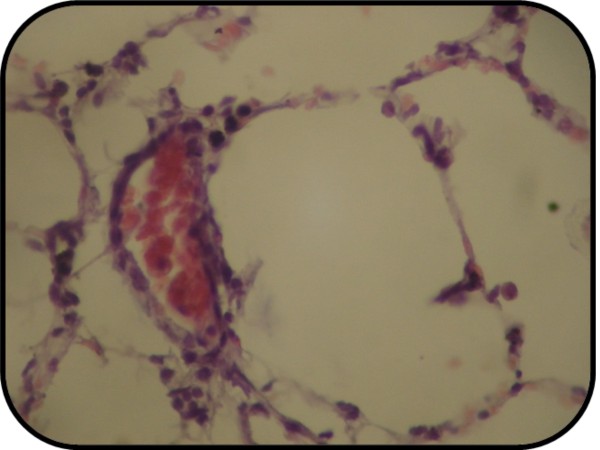
**Photomicrograph of lung section with mild injury**. The section stained with Haematoxylin and Eosin (X 40).

Treatment of rats with MK-886 ameliorated the lung injury significantly (*P *< 0.05) as compared with induced untreated group and the total score mean of this group showed mild lung injury (Figure 11). 16.7% of the group had normal histopathological appearance and 83.3% of the group had mild lung injury as shown in table 5.

## Discussion

The present study demonstrates that HS causes ALI, as evidenced by biochemical and histologic changes. MK-886 prevented the biochemical changes and protected the lung morphology after HS. Although leukotrieneshave been known to be associated with the I/R injury in other tissues, including intestine [24] kidney [25], myocardium [26] and liver [27], there are only a few studies describing the correlation between hemorrhagic shock-induced lung injury and 5-lipoxygenase pathway products, where two studies demonstrated that the 5-lipoxygenase pathway products meditate acute lung injury following hemorrhagic shock [28,29]. And it has been demonstrated that LTB4 levels were significantly increased in the rat lungs following T/HS [30]. Studies in humans confirm elevated levels of LTB_4_, LTC_4_, LTD_4 _in BAL, pulmonary edema fluid, and plasma in patients with ALI compared with "at-risk" group or those with hydrostatic edema [31,32]. In the present study a significant increase in BALF leukotriene (LTB_4 _& LTC_4_) levels were found in the shocked rats as compared with sham group. The increased leukotriene level in shocked rats might be due to the associated splanchnic I/R, which activates gut PLA_2_-mediated release of AA into the lymph where it is delivered to the lungs [33]. Arachidonic acid is a biologically active lipid released from the cellular membrane by PLA_2 _that can engage the LTB_4 _receptor and initiate LTB_4 _production with autocrine effects [34] . Arachidonic acid also promotes 5-lipoxygenase translocation to the nucleus, a key step in leukotrienes production [35]. Additionally, it is known that ischemia elevates cytosolic calcium concentration, which in turn elevates PLA_2 _and lipoxygenase activity, generating leukotrienes. Furthermore, increased leukotriene level might be due to the leukocytes accumulated in the lungs as observed in the histological section of the shocked rat lung where activated neutrophils following hemorrhagic shock are capable of releasing cytotoxic products including leukotrienes, and the intrinsic 5-lipoxygenase activity is required for neutrophil adherence and chemotaxis and neutrophil-mediated lung injury [36]. In addition to neutrophils, alveolar macrophages and circulating macrophages aggravate lung injury and alveolar neutrophil sequestration in hemorrhagic shock [37] and might contribute to further release of leukotrienes. In this study we have demonstrated that treatment with MK-886 appeared to have a significant decrease in BALF leukotrienes (LTB_4 _& LTC_4_) level in the shocked rats in comparison with the induced untreated group. It is reported that selective inhibition of leukotriene biosynthesis by MK-886 prevents postischemic leukotrienes accumulation and the microcirculatory changes after I/R in the striated muscle in vivo [14]. Furthermore, MK-886 was found to be a potent and specific inhibitor of both LTB_4 _and LTC_4 _synthesis in human phagocytes [9,38].

Hemorrhagic shock is considered as an insult frequently leading to systemic inflammatory response syndrome including the systemic release of proinflammatory cytokines which is central in the inflammatory response. Previous studies have shown that levels of IL-6 and TNF-α significantly increased following trauma-hemorrhage and remain elevated for several hours [39]. The results in present study are consistent with that reported by **Vincenzi *et al***. [40] Who found that a significant increase in the TNF-α and IL-6 levels in shocked rats in comparison with sham group. Activated inflammatory cells, especially macrophages and neutrophils have been shown to play a pivotal role in the propagation of SIRS following resuscitated shock and could be considered the main source of inflammatory cytokines including TNF-α and IL-6. In this study MK-886 significantly reduced the elevation of IL-6 and TNF-α level in the shocked rats as compared with induced untreated group suggesting that MK-886 has protective effect in hemorrhagic shock-induced acute lung injury. Inhibition of endogenous CysLT production by MK-886 significantly attenuated the generation of TNF-α by mast cells activated by FcεRI cross-linkage [41]. MK-886 pretreatment attenuated subsequent pulmonary expression of TNF- α in a mouse model of bronchial inflammation and hyperreactivity [42]. LTB_4 _augments IL-6 production in human monocytes by increasing both IL-6 gene transcription and mRNA stabilization [43,44]. activation of NF-κB and NF-IL-6 transcriptional factors may be important in this enhancement of IL-6 release [44]. Furthermore, TNF-α production is enhanced by LTC_4 _and LTD_4 _[45]. So that, inhibition of LTB_4 _and CysLTs synthesis by MK-886 might result in lowering TNF-α and IL-6 levels.

Through examination of metabolic processes, GSH has been shown to be important in host defenses against oxidative stress [46] . Another important agent showing oxidative stress is MDA, a marker of free radical activity [4]. It was reported that oxidative stress significantly elevated MDA levels and reduced GSH levels [47]. Oxidative stress has been implicated as an important cause of HSR pathogenesis [2,46]. The result in present study are consistent with other study who found that a significant increase in lung MDA level and significant decrease in lung GSH level were found in hemorrhagic shock group as compared to sham group in a rat model of hemorrhagic shock-induced acute lung injury [18]. In this study MK-886 significantly reduced the elevation of lung MDA level and significantly elevates the lung GSH level in the shocked rats as compared with induced untreated group suggesting that MK-886 has protective effect in hemorrhagic shock-induced oxidative injury of the lung. There is no data available about the effect of MK-886 on oxidative lung injury in HS. But they found that MK-886 significantly reduces hepatic and intestinal MDA level and elevates GSH level in these organs in rats that underwent hepatic I/R model and anti-inflammatory properties and inhibition of lipid peroxidation by MK-886 could be protective for these organs in I/R injury [18]. The antioxidant effect of MK-886 might be largely due to its inhibitory action on leukotrienes synthesis.

In the present study a significant increase in the BALF total protein level was found in the shocked rats as compared with sham group, suggesting that hemorrhagic shock induces lung injury in rats. Increased protein concentration in BALF is an important marker of damage to the alveolar-capillary barrier of lung. Furthermore, the increase in BALF total protein concentration may be due to increased lung permeability and lung edema during acute lung injury [48]

The acute phase of ALI and ARDS is characterized by the influx of protein-rich edema fluid into the air spaces as a consequence of increased permeability of the alveolar-capillary barrier [49]. As previously reported, T/HS caused lung injury as reflected in increased permeability to Evans blue dye, BALF protein levels and the BALF to plasma protein ratio [50,51]. Two studies showed that hemorrhagic shock significantly increases BALF total protein in the rats and mice [20,29]. CysLTs mediate increased permeability leading to leukocyte extravasation, plasma exudation and edema[52, 53, and 54]. Furthermore, LTB_4 _increases the expression of CD11b/CD18 β2-integrin (Mac-1) on neutrophils, which can facilitate neutrophil adherence and migration [55] and enhanced leukocyte adhesivity accounts for capillary obstruction after I/R [56]. T/HS lymph induces an increase in endothelial permeability by triggering the release of IL-6 [57] . It has been demonstrated that IL-6 is an important autocrine factor produced by endothelial cells that contributes to the increase in endothelial permeability during hypoxia [58]. Free radicals are implicated to damage biomembranes, thereby compromising cell integrity and function [59] . Besides increasing pulmonary arterial pressure [60], the free radical production under hypoxic environment may cause oxidative injury of the endothelium [61], resulting in increased pulmonary capillary permeability. In this study treatment with MK-886 appeared to have a significant decrease in BALF total protein level in the shocked rats in comparison with the induced untreated group. MK-886 has been shown to reduce the extravasation of plasma [13] and prevent the leukocyte adhesion to the endothelium [14] in experimental animals. It was demonstrated that treatment of mice with MK-886 significantly abolished the increase in the BALF total protein level in acute lung injury following hemorrhagic shock [29].

Morphologically, there was a statistically significant difference between induced untreated group and sham group and the total score mean of the HS group shows moderate lung injury. 66.7% of the HS group had moderate lung injury and 33.3% had severe lung injury. Treatment of rats with MK-886 ameliorates the lung injury significantly as compared with induced untreated group and the total score mean of the control group shows mild lung injury. Although there is no data available about the protective effect of MK-886 on the lung parenchyma in HS rats, but they found that MK-886 significantly reduces the histological changes in the liver and small intestine of rats that underwent hepatic I/R model (15). MK-886 was able to reduce the cortical infarct size by 30% in a model of focal cerebral ischemia in rats [62] . Furthermore, a separate research work found that treatment of rats with MK-886 reduces brain lesion volume in experimental traumatic brain injury model [63].

## Competing interests

The authors participated in the design of the study and performed the statistical analysis declare that they have no competing interests.

## Authors' contributions

FG carried out the surgical experimental work and gives the outline of research. NR participated in the design of the study and performed the statistical analysis and supervised main skeleton. AM participated in the sequence alignment and drafted the manuscript and did all the biochemical and histopathological tests.

All authors read and approved the final manuscript.
